# SDADB: a functional annotation database of protein structural domains

**DOI:** 10.1093/database/bay064

**Published:** 2018-06-28

**Authors:** Cheng Zeng, Weihua Zhan, Lei Deng

**Affiliations:** 1 School of Software, Central South University, Changsha 410075, China; 2 School of Electronics and Computer Science, Zhejiang Wanli University, Ningbo 315100, China; 3 Shanghai Key Lab of Intelligent Information Processing, Shanghai 200433, China

## Abstract

Annotating functional terms with individual domains is essential for understanding the functions of full-length proteins. We describe SDADB, a functional annotation database for structural domains. SDADB provides associations between gene ontology (GO) terms and SCOP domains calculated with an integrated framework. GO annotations are assigned probabilities of being correct, which are estimated with a Bayesian network by taking advantage of structural neighborhood mappings, SCOP-InterPro domain mapping information, position-specific scoring matrices (PSSMs) and sequence homolog features, with the most substantial contribution coming from high-coverage structure-based domain-protein mappings. The domain-protein mappings are computed using large-scale structure alignment. SDADB contains ontological terms with probabilistic scores for more than 214 000 distinct SCOP domains. It also provides additional features include 3D structure alignment visualization, GO hierarchical tree view, search, browse and download options.

Database URL: http://sda.denglab.org

## Introduction

A protein domain is a conserved and functional unit of a protein that can fold independently and has distinct functions. Most proteins consist of one or several domains. A unique domain may appear in a variety of different proteins that capture specific functions. Usually, specific functions of protein domains are highly independent, and they are, in many cases, conserved across species (1). For example, the catalytic domain of serine/threonine/tyrosine protein kinases is highly conserved from *E. coli* to human containing the catalytic function, and shares conserved catalytic regions with both serine/threonine and tyrosine protein kinases (2). The N-terminal of the catalytic domain has been shown to be involved in ATP binding, while the central part of the catalytic domain plays important roles in the catalytic activity of the enzyme (3, 4). A broad range of approaches has been developed to the problem of automatically identifying domain regions in protein sequences based on some degree of relatedness shared between domain sequences. InterPro (5, 6) is the widely used sequence-based domain database, which collates important resources for protein domain classifications: Pfam (7), CATH-Gene3D (8), SMART (9), ProDom (10), SUPERFAMILY (11) and PROSITE (12). The Conserved Domain Database (CDD) (13) maintains domain annotations for sequences. It produces representative sequence fragments, which are in agreement with domain boundaries as observed in protein 3D structure. A more reliable way to assign structures to the domain families is using the structural information. As the widely used hierarchical classification scheme of proteins, SCOP (14) groups protein domains into Class, fold, superfamily and family according to structural and evolutionary relationships (15). The current version of SCOPe version 2.06 (16, 17) contains over 240 000 structural domains.

Assigning ontological terms to specific domains are important for fully understanding functions of proteins. Gene ontology (GO) has been a de facto standard for describing gene and protein function (18, 19). It arranges in a directed acyclic graph and discriminates between molecular function and biological process, as well as subcellular localization. The GO terms in top levels describe general functions such as catalytic activity and binding. While deeper GO terms in the hierarchy represent more specific functions. For sequence-based domains, a few have been manually annotated with GO terms, and several computational prediction methods have been developed. The InterPro2GO mapping (20) is curated manually by the InterPro team, who compare InterPro and protein entries, check the statistic and conservation information, and assign most appropriate and specific GO terms to the InterPro domain. The Pfam2GO mapping is subsequently created by mapping InterPro domains to Pfam domains. Forslund and Sonnhammer (21) developed a probabilistic model to predict the relationship between multiple Pfam domain and annotation GO terms. Rentzsch and Orengo (22) use domain functional families (FunFams) to predict the functions of whole proteins. They group domain sequences into FunFams based on the GO annotations and associate the FunFams with GO terms probabilistically.

Although sequence-based domain annotation and domain-centric protein function prediction have been extensively studied, predicting functions for protein structural domains is, even more, difficult given the lack of comprehensive structural domain information for proteins. Only a few previous efforts have been performed to computationally predict structural domain functions (11, 23, 24). The SUPERFAMILY database (11) contains SCOP domain architecture and classification assignments to sequences at the superfamily level by using hidden Markov models. Based on the sequence homology to SCOP structural domain mapping in SUPERFAMILY, the dcGO database (24) provides GO annotations for SCOP domains in a probabilistic framework at the superfamily and family levels. Daniel and Florencio (23) proposed a scop2go approach, which annotates SCOP domains with molecular function GO terms based on the fold distribution of PDB structures associated with given GO terms. Although these resources are valuable, they only have coarse-grained level function annotation and are largely incomplete in that many domains are still not annotated.

Recently, we proposed a functional annotation approach for structural domains (SDA) that is largely based on 3D structure-based domain-protein mappings (25). We used a Bayesian network to integrate heterogeneous information: (i) protein-to-domain mappings calculated using all-against-all structural alignment of SCOP domains and protein structures from the PDB database; (ii) SCOP-to-InterPro domain mappings calculated using the InterProScan software; (iii) SVM models generated based on the position-specific scoring matrix (PSSM) profiles; (iv) sequence homologs mapped to SCOP domains using a Bayesian network. We showed the advantages of integrating large-scale structure-based mappings and other heterogeneous information sources for structural domain function prediction.

Here, we present the SDA database, which provides domain GO annotations predicted from our integrated method, and also includes links to other databases. The server allows users to query functional annotations for input proteins or domains. The results can be visualized in an interactive 3D viewer and a tree viewer. SDADB is available at http://sda.denglab.org.

## Methods and data sources

**Figure 1.**
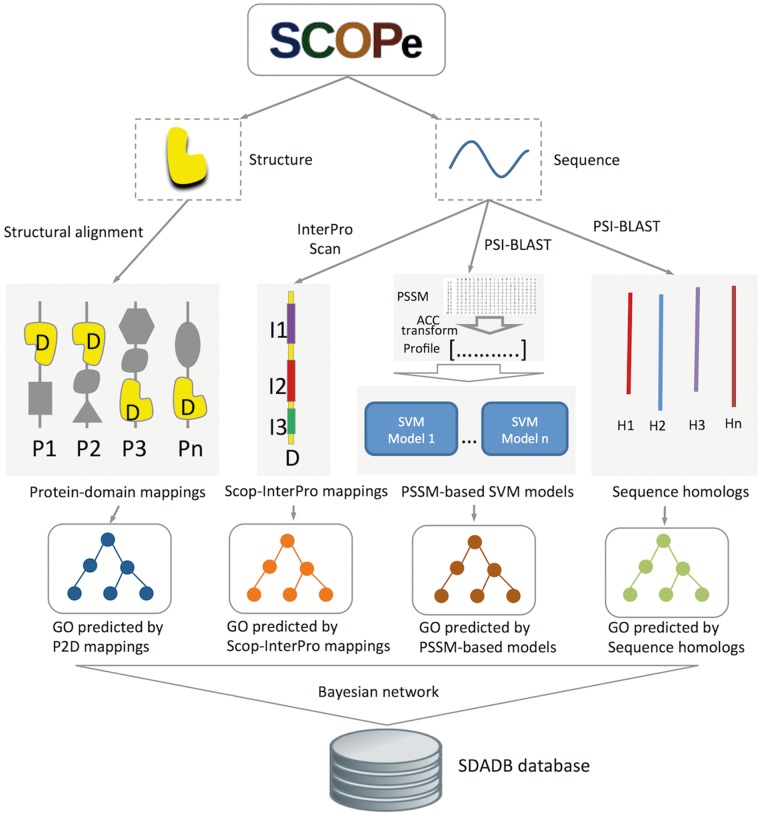
Flowchart of SDADB construction. For each domain in the SCOPe database, GO annotations are predicted with the four component methods: (i) GO annotations predicted using P2D mappings: protein-SCOP domain mappings are calculated by large-scale structure alignment, then the probability that a domain annotated by a specific function is computed; (ii) GO annotations predicted using Scop-InterPro mappings: we use InterProScan to search InterPro domains for the SCOP domain, and transfer the annotations of these InterPro domains in the InterPro2GO database to the target SCOP domain; (iii) GO annotations predicted using PSSM profiles: SVM models for GO function annotation are trained with fixed length of PSSM vectors, which are calculated using ACC transformation; (iv) GO annotations predicted using sequence homologs: we transfer the GO annotations of the sequence homologs in UniProt-GOA to the target SCOP domain. Finally, the SDADB database is built by integrating the outputs of the four component methods with a Bayesian network.

Structural domains are downloaded from SCOPe version 2.06 (16, 17). GO annotations for the SCOP domains are generated by our structure-based integrative function prediction approach that combines structural mappings with other sequence and evolutionary clues (25). A detailed illustration of the data sources and framework is shown in Figure 1. Briefly, for a query SCOP domain, GO annotations are predicted with four component methods (structure-based, InterPro-based, PSSM-based and sequence homology-based methods). A probability for each annotation is calculated using a Bayesian network trained on a dataset of SCOP domains (ending in dash) generated from single-domain proteins.

### GO annotations predicted using protein-domain structural mappings

We use a structure alignment algorithm (26, 27) to search structural neighbors for SCOP domains against the PDB database, and obtain a significant number of protein-domain (P2D) mappings. The structural similarity between proteins and domains is measured by protein structure distance (PSD) (26). The PSD score integrates the RMSD (root mean square deviation) and the secondary structural alignment score to measure the similarity of two structures and is applicable both when two structures are very similar, and when they are very different. Lower PSD score corresponds to a good fit or better alignment between the structures. A protein is defined as the structural neighbor of a SCOP domain when the PSD score is lower than 0.1. We assign GO annotations to SCOP domains based on the assumption that the most populated SCOP domain in the mappings corresponds to the structural neighbor proteins which are responsible for the function (25). The GO annotations of proteins are extracted from the PDB-GOA database (28). The probabilities of transferring protein function annotations to SCOP domains are computed as shown in the following equation:

$$P_{1}(g|d)=\frac{P(d,g)}{P(d)},$$

where *d* is a SCOP domain, *g* denotes a GO term, *P*(*d*, *g*) is the number of PDB proteins containing domain *d* and having function *g*, *P*(*d*) is the number of PDB proteins containing domain *d* calculated on the P2D mapping data.

### GO annotations predicted using Scop-InterPro domain mappings

We use the InterProScan tool (6) to search the InterPro domains (5) against the SCOP sequences. GO terms of InterPro domains in the InterPro2GO database (28) are transferred to the corresponding SCOP domains. The probability of a GO term (*g*) assigned to the SCOP domain is calculated based on the number of InterPro domains that have the function:

$$P_{2}(g)=\frac{1}{n}\overset{n}{\underset{i=1}{\sum^{}}}I(g),$$

where *n* is the number of InterPro domains owned by the SCOP domain. If an InterPro domain has the function *g*, *I*(*g*) is 1; otherwise 0.

### GO annotations predicted using PSSM profiles

PSSM (29–32) is a highly informative representation of protein sequences and is widely utilized in many applications. We use PSI-BLAST (33) to calculate PSSM profiles based on the NCBI NR database (34). The auto-cross covariance (ACC) transformation (35) is used to transform the PSSM profiles into fixed-length vectors. For a domain sequence, auto covariance (AC) describes the average interactions between residues, a certain distance (*l*) apart throughout the whole sequence. For a descriptor (one of the 20 basic residue types), the AC variable is calculated as:

$$AC=\frac{1}{DL-l}\overset{DL-l}{\underset{i=1}{\sum^{}}}(X_{i}-\bar{X})(X_{i+l}-\bar{X}),$$

where *l* denotes the distance between one residue and its neighbor, *DL* is the length of domain sequence, *X_i_* is the PSSM score of the descriptor at position *i*, $\overset{-}{X\;}$is the average score for the descriptor along the whole sequence. We use the AC variables to transform the numerical PSSM vectors of SCOP domain sequences into uniform matrices with the distance *l *=* *10. Based on the PSSM vectors, we build SVM classifiers for each GO term. The probability score of a GO term (*g*) assigned to a SCOP domain is estimated based on the output of the SVM classifier by a sigmoid function:

$$P_{3}(g)\approx \;\frac{1}{1+e^{Af(g)+B}},$$

where *f*(*g*) is the SVM output score, *A* and *B* are estimated using the method of Lin *et al.* (36).

### GO annotations predicted using sequence homologs

We also used PSI-BLAST to search sequence homologs for SCOP domains against the UniProt database. We only select the sequence homologs with alignment coverage >60%. The GO annotations of sequence homologs are obtained from the UniProt-GOA database (37). We use the sequence homolog’s *E*-value (*E*) to estimate the weight of its GO term assigned to the query SCOP domain. The probability for a GO term (*g*) assigned to the query domain is the sum of weights of sequence homologs that have the GO annotation in UniProt-GOA:

$$P_{4}(g)=\sum_{i=1}^{n}\frac{-\log\left(E_{i}\right)+b}{\sum_{j=1}^{n}\left\{-\log\left(E_{j}\right)+b\right\}}\cdot I(g)$$

where *n* is the number of sequence homologs, *b* is a constant of log(10). If the sequence homolog has the function *g*, *I*(*g*) is 1; otherwise 0.

### Integration of the four component methods

We combine the output scores of the four component approaches using a Bayesian network (38). The Bayesian network represents the joint probability distribution of multiple variables and is especially suitable for integrating heterogeneous data sources. We split the probability scores of the four component methods into individual bins. The likelihood ratio (LR) (39) for any bin is calculated as the ratio of the odds of a GO annotation to be true or false after and before knowing it is in this bin. The LR represents the increase of the chance that a GO prediction method with a particular set of scores corresponds to a positive GO annotation, compared with a random GO assignment. The final probability score is calculated by integrating the LR of the four component methods:

$$LR=\prod_{i}^{N}LR\left(f_{i}\right),$$

where *f* is the component method.

The final predicted GOA-SCOP (GO annotation for SCOP domains) data are stored in an MYSQL database. The website is developed using Perl, JavaScript, jQuery (AJAX), CSS and HTML5, and is deployed on an Apache web server. The BioJava (40) and JSmol (41) provide visuals of the P2D alignment. D3 (D3.js) (42) is used for visualizing GO hierarchical tree.

## Web server interface

**Figure 2.**
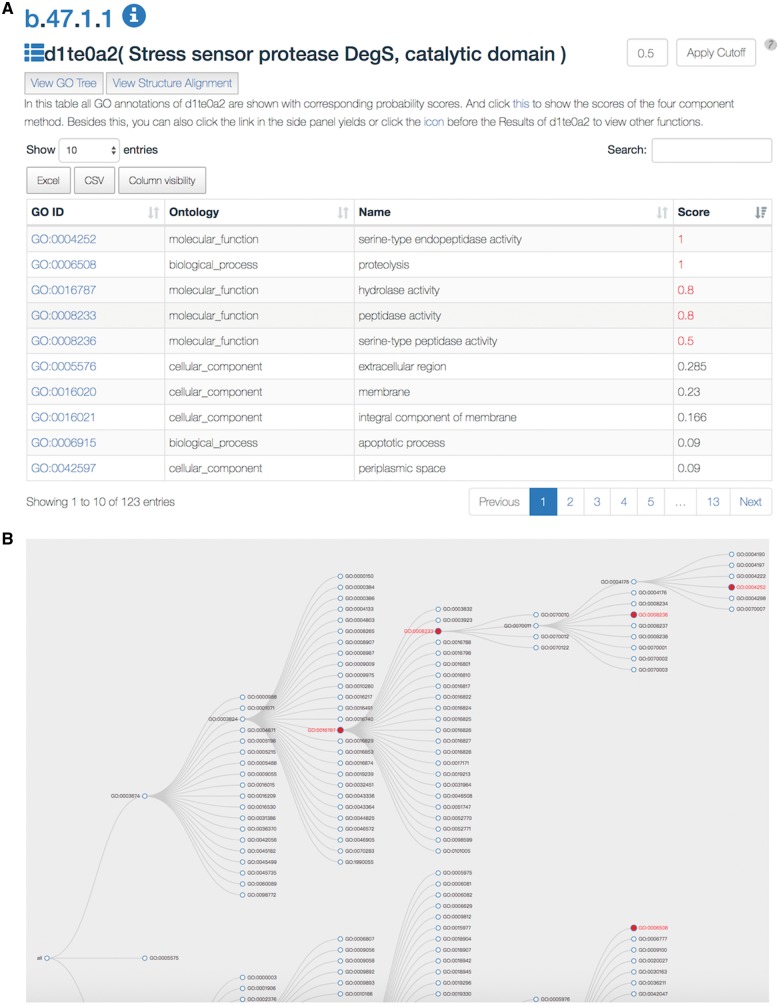
A snapshot of the SDADB web interface. (A) The GO annotations of a query domain are listed. (B) The GO tree view shows the hierarchical architecture of GO for the query domain.

The SDADB database can be queried through the protein/domain accession number (e.g. 1te0/d1te0a2) or protein/domain name (e.g. Stress sensor protease DegS). The server will return a list of GO annotations with corresponding confidence scores (Figure 2A). The detailed annotation information, including GO accession ID, GO type, GO name and associated score, are shown in the table. The associated score denotes the probability of the SCOP domain certain having the GO function. The higher the score is, the more likely the SCOP domain has the function. The default cut-off of the associated score is 0.5. The GO annotations with scores over the cut-off are colored in red. The users can change the threshold according to their own needs. Also, users can search GO annotations in the results and download the detailed results in Excel or CSV file.

**Figure 3.**
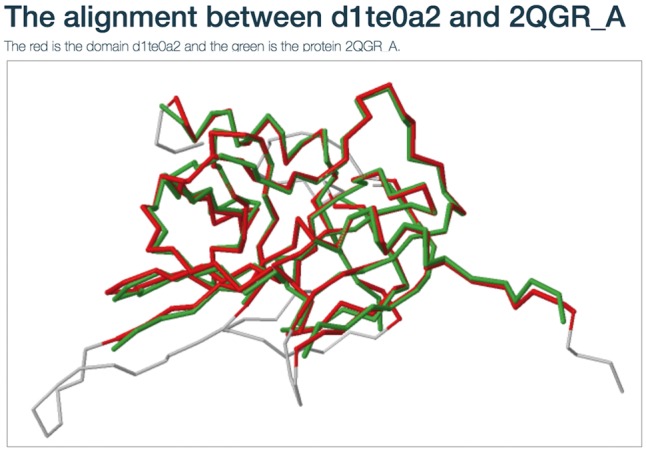
The structure alignment view for the domain-protein mappings.

Users can view the annotations by clicking the ‘view GO tree’ button, which shows the hierarchical architecture of GO (Figure 2B). Users can expand or collapse the term nodes. The red nodes in the tree are annotated GO terms of the target SCOP domain. Users can view the GO name by putting the mouse over the node. Another unique feature is the visualization of structure alignment for the domain-protein mappings, which constitutes a major contribution to the function prediction. Users can choose to view the alignment between the target domain and its structural neighbors in 3D view (Figure 3).

## Results

To evaluate the accuracy of domain functional annotations, we use the dataset obtained from GOA-PDB version 201010 in training and the independent test set from GOA-PDB version 201311 excluding those in GOA-PDB version 201010 for testing. Proteins of the test set that have >90% sequence identity to the proteins in the training set are removed. We use the precision–recall curve and maximum *F*-measure (*F*_max_) to measure the overall performance. The precision–recall curve shows the trade-off between precision and recall for different thresholds. A high area under the precision–recall curve denotes high overall performance. *F*-measure considers both the precision and the recall of the GO prediction results of SCOP domains. It is calculated as the harmonic average of the precision and recall. Maximum *F*-measure (*F*_max_) is the maximum value of the *F*-measure over a varying threshold. The coverage is computed by dividing the number of domains with predicted GO annotations by the total number of domains in SCOPe 2.06. For detailed descriptions of the datasets and performance measures, see Reference (25).

**Figure 4.**
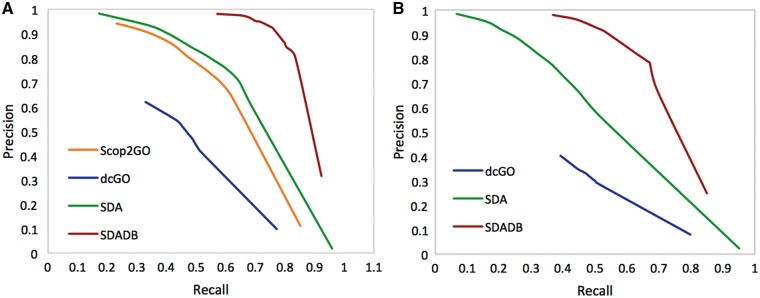
Precision–recall curve of SDADB versus existing methods for molecular function (A) and biological process (B).

We compare our SDADB database with the four component methods, including structure alignment-based method (Str), Interpro domain-based method (IPR), PSSM profile-based method (PSSM) and sequence homolog-based approach (Seq). The results are summarized in Table 1. We observe that the combined SDADB significantly outperforms the four component methods, with a maximum *F*-score of 0.833 for MF (molecular function), a maximum *F*-score of 0.723 for BP (biological process) and a maximum *F*-score of 0.809 for CC (cellular component). For the coverage, SDADB has GO annotations for most SCOP domains (92.3%). We also compare SDADB with other state-of-the-art approaches on the independent test dataset. As shown in Figure 4, it is clear that SDADB significantly outperforms SDA and other methods for both MF and BP.

**Table 1.** Prediction performance comparison of SDADB with the four component methods

Methods	Coverage	*F*max (MF)	*F*_max_ (BP)	*F*_max_ (CC)
Str	0.802	0.806	0.690	0.767
Blast	0.918	0.755	0.616	0.687
PSSM	0.509	0.491	0.364	0.507
IPR	0.555	0.711	0.508	0.263
SDADB	**0.923**	**0.833**	**0.723**	**0.809**

## Conclusion

The SDADB database provides large-scale detailed GO annotations at the structural domain level. In contrast to the approaches based on sequence and homology information, an advantage of SDADB is that the method integrates structural neighborhood features together with a variety of heterogeneous information, including SCOP-InterPro domain mapping information, PSSMs and sequence homolog features. The SDADB database now contains 3 482 316 GO annotations for 211 282 SCOP domains with a probability >0.1. Of these, 1 479 652 annotations for 204 948 domains have a probability >0.5. Also, SDADB provides P2D mappings for over 191 060 PDB structures. The vast amount of P2D and domain-function mapping data in the SDADB database can help to investigate the functions of full-length proteins since domains are functional units of proteins. The database will also give valuable insights into protein domain evolution, which are not only likely to be fascinating but will also ultimately improve the power and accuracy of protein function prediction approaches.

It is worth pointing out that some common and multifunctional domains may be not well annotated since the presence of a common domain in several proteins does not necessarily imply that these proteins have the same function. Future developments will focus on combining more informative clues and analyzing tools. We also expect the interested user will be able to use the resources provided in the SDADB database as a basis for new efforts on expanding the functional space for both domains and full-length proteins.

## Availability

The SDADB database is freely available at http://sda.denglab.org/.

## Funding

This work was supported by National Natural Science Foundation of China (61672541); Natural Science Foundation of Hunan Province (2017JJ3287); Natural Science Foundation of Zhejiang (LY13F020038); Fundamental Research Funds for the Central Universities of Central South University (2017zzts727) and Shanghai Key Laboratory of Intelligent Information Processing (IIPL-2014-002). 

*Conflict of interest*. None declared.

## References

[1] Marcotte E.M., Pellegrini M., Ng H.-L. et al (1999) Detecting protein function and protein-protein interactions from genome sequences. Science, 285, 751–753. 10427000 10.1126/science.285.5428.751

[2] Hanks S.K., Quinn A.M., Hunter T. (1988) The protein kinase family: conserved features and deduced phylogeny. Science, 241, 42–52. 3291115 10.1126/science.3291115

[3] Knighton D.R., Zheng J., Lynn F. et al (1991) Crystal structure of the catalytic subunit of cyclic adenosine monophosphate-dependent protein kinase. Science, 407–414. 1862342 10.1126/science.1862342

[4] Zou Q., Chen W., Huang Y. et al (2013) Identifying multi-functional enzyme by hierarchical multi-label classifier. J. Comput. Theor. Nanosci., 10, 1038–1043.

[5] Finn R.D., Attwood T.K., Babbitt P.C. et al (2017) InterPro in 2017—beyond protein family and domain annotations. Nucleic Acids Res., 45, D190–D199. 27899635 10.1093/nar/gkw1107 PMC5210578

[6] Zdobnov E.M., Apweiler R. (2001) InterProScan—an integration platform for the signature-recognition methods in InterPro. Bioinformatics, 17, 847–848. 11590104 10.1093/bioinformatics/17.9.847

[7] Bateman A., Coin L., Durbin R. et al (2004) The Pfam protein families database. Nucleic Acids Res., 32, D138–D141. 14681378 10.1093/nar/gkh121 PMC308855

[8] Sillitoe I., Lewis T.E., Cuff A. et al (2015) CATH: comprehensive structural and functional annotations for genome sequences. Nucleic Acids Res., 43, D376–D381. 25348408 10.1093/nar/gku947 PMC4384018

[9] Letunic I., Doerks T., Bork P. (2012) SMART 7: recent updates to the protein domain annotation resource. Nucleic Acids Res., 40, D302–D305. 22053084 10.1093/nar/gkr931 PMC3245027

[10] Bru C., Courcelle E., Carrère S. et al (2005) The ProDom database of protein domain families: more emphasis on 3D. Nucleic Acids Research, 33, D212–D215. 15608179 10.1093/nar/gki034 PMC539988

[11] Oates M.E., Stahlhacke J., Vavoulis D.V. et al (2015) The SUPERFAMILY 1.75 database in 2014: a doubling of data. Nucleic Acids Res., 43, D227–D233. 25414345 10.1093/nar/gku1041 PMC4383889

[12] Hulo N., Bairoch A., Bulliard V. et al (2006) The PROSITE database. Nucleic Acids Res., 34, D227–D230. 16381852 10.1093/nar/gkj063 PMC1347426

[13] Marchler-Bauer A., Derbyshire M.K., Gonzales N.R. et al (2015) CDD: nCBI's conserved domain database. Nucleic Acids Res., 43, D222–D226. 25414356 10.1093/nar/gku1221 PMC4383992

[14] Murzin A.G., Brenner S.E., Hubbard T., Chothia C. (1995) SCOP: a structural classification of proteins database for the investigation of sequences and structures. J. Mol. Biol., 247, 536–540. 7723011 10.1006/jmbi.1995.0159

[15] Wei L., Zou Q. (2016) Recent progresses in machine learning-based methods for protein fold recognition. Int. J. Mol. Sci., 17, 2118. 10.3390/ijms17122118 PMC5187918 27999256

[16] Chandonia J.-M., Fox N.K., Brenner S.E. (2017) SCOPe: manual curation and artifact removal in the structural classification of proteins–extended database. J. Mol. Biol., 429, 348–355. 27914894 10.1016/j.jmb.2016.11.023 PMC5272801

[17] Fox N.K., Brenner S.E., Chandonia J.-M. (2014) SCOPe: structural classification of proteins—extended, integrating SCOP and ASTRAL data and classification of new structures. Nucleic Acids Res., 42, D304–D309. 24304899 10.1093/nar/gkt1240 PMC3965108

[18] Zhang J., Zhang Z., Wang Z. et al (2018) Ontological function annotation of long non-coding RNAs through hierarchical multi-label classification. Bioinformatics, 34, 1750–1757. 29293953 10.1093/bioinformatics/btx833

[19] Zhang Z., Zhang J., Fan C. et al (2017) KATZLGO: large-scale prediction of LncRNA functions by using the KATZ measure based on multiple networks. IEEE/ACM Trans. Comput. Biol. Bioinform., doi: 10.1109/TCBB.2017.2704587. 10.1109/TCBB.2017.2704587 28534780

[20] Burge S., Kelly E., Lonsdale D. et al (2012) Manual GO annotation of predictive protein signatures: the InterPro approach to GO curation. Database, 2012, doi: 10.1093/database/bar068. 10.1093/database/bar068 PMC3270475 22301074

[21] Forslund K., Sonnhammer E.L.L. (2008) Predicting protein function from domain content. Bioinformatics, 24, 1681–1687. 18591194 10.1093/bioinformatics/btn312

[22] Rentzsch R., Orengo C.A. (2013) Protein function prediction using domain families. BMC Bioinformatics, 14, S5. 10.1186/1471-2105-14-S3-S5 PMC3584934 23514456

[23] Lopez D., Pazos F. (2009) Gene ontology functional annotations at the structural domain level. Proteins Struct. Funct. Bioinform., 76, 598–607. 10.1002/prot.22373 19241468

[24] Fang H., Gough J. (2013) dcGO: database of domain-centric ontologies on functions, phenotypes, diseases and more. Nucleic Acids Res., 41, D536–D544. 23161684 10.1093/nar/gks1080 PMC3531119

[25] Deng L., Chen Z. (2015) An integrated framework for functional annotation of protein structural domains. IEEE/ACM Trans. Comput. Biol. Bioinform., 12, 902–913. 26357331 10.1109/TCBB.2015.2389213

[26] Yang A.-S., Honig B. (2000) An integrated approach to the analysis and modeling of protein sequences and structures. I. Protein structural alignment and a quantitative measure for protein structural distance. J. Mol. Biol., 301, 665–678. 10966776 10.1006/jmbi.2000.3973

[27] Zhang Q.C., Petrey D., L., Deng L.Q., Shi Y. et al (2012) Structure-based prediction of protein-protein interactions on a genome-wide scale. Nature, 490, 556. 23023127 10.1038/nature11503 PMC3482288

[28] Camon E., Magrane M., Barrell D. et al (2004) The gene ontology annotation (GOA) database: sharing knowledge in uniprot with gene ontology. Nucleic Acids Res., 32, 262D–D266. 10.1093/nar/gkh021 PMC308756 14681408

[29] Jones D.T. (1999) Protein secondary structure prediction based on position-specific scoring matrices. J. Mol. Biol., 292, 195–202. 10493868 10.1006/jmbi.1999.3091

[30] Fan C., Liu D., Huang R. et al PredRSA: a gradient boosted regression trees approach for predicting protein solvent accessibility. BMC Bioinformatics, 17, 8. 26818760 10.1186/s12859-015-0851-2 PMC4895273

[31] Wei L., Tang J., Zou Q. (2017) Local-DPP: an improved DNA-binding protein prediction method by exploring local evolutionary information. Inform. Sci., 384, 135–144.

[32] Pan Y., Wang Z., Zhan W. et al (2018) Computational identification of binding energy hot spots in protein-RNA complexes using an ensemble approach. Bioinformatics, 34, 1473–1480. 29281004 10.1093/bioinformatics/btx822

[33] Altschul S.F., Madden T.L., Schäffer A.A. et al (1997) Gapped BLAST and PSI-BLAST: a new generation of protein database search programs. Nucleic Acids Res., 25, 3389–3402. 9254694 10.1093/nar/25.17.3389 PMC146917

[34] Li W., Godzik A. (2006) Cd-hit: a fast program for clustering and comparing large sets of protein or nucleotide sequences. Bioinformatics, 22, 1658–1659. 16731699 10.1093/bioinformatics/btl158

[35] Guo Y., Yu L., Wen Z. et al (2008) Using support vector machine combined with auto covariance to predict protein–protein interactions from protein sequences. Nucleic Acids Res., 36, 3025–3030. 18390576 10.1093/nar/gkn159 PMC2396404

[36] Lin H.-T., Lin C.-J., Weng R.C. (2007) A note on Platt’s probabilistic outputs for support vector machines. Mach. Learn., 68, 267–276.

[37] Dimmer E.C., Huntley R.P., Alam-Faruque Y. et al (2012) The UniProt-GO annotation database in 2011. Nucleic Acids Res., 40, D565–D570. 22123736 10.1093/nar/gkr1048 PMC3245010

[38] Friedman N., Geiger D., Goldszmidt M. (1997) Bayesian network classifiers. Mach. Learn., 29, 131–163.

[39] Jansen R., Yu H., Greenbaum D. et al (2003) A Bayesian networks approach for predicting protein-protein interactions from genomic data. Science, 302, 449–453. 14564010 10.1126/science.1087361

[40] Prlić A., Yates A., Bliven S.E. et al (2012) BioJava: an open-source framework for bioinformatics in 2012. Bioinformatics, 28, 2693–2695. 22877863 10.1093/bioinformatics/bts494 PMC3467744

[41] Hanson R.M., Prilusky J., Renjian Z. et al (2013) JSmol and the Next-generation web-based representation of 3D molecular structure as applied to proteopedia. Israel J. Chem., 53, 207–216.

[42] Ogievetsky V., Heer J., Bostock J. (2011) D3 data-driven documents. IEEE Trans. Vis. Comput. Graph, 17, 2301–2309. 22034350 10.1109/TVCG.2011.185

